# Cross-Cultural Adaptation and Psychometric Validation of the Constipation Assessment Scale among Chinese Adult Psychiatric Patients

**DOI:** 10.3390/ijerph20032703

**Published:** 2023-02-02

**Authors:** Wai Kit Wong, Jing Qin, Daniel Bressington, Wing Fai Yeung, Ning Liu, Bryan Ying Wai Ho, Surui Liang, Yan Li

**Affiliations:** 1 School of Nursing, Tung Wah College, Hong Kong SAR, China; 2 School of Nursing, The Hong Kong Polytechnic University, Hong Kong SAR, China; 3 Faculty of Health, Charles Darwin University, Ellengowan Drive, Darwin, NT 0909, Australia; 4 School of Nursing, Zunyi Medical University, Zunyi 563002, China

**Keywords:** constipation assessment scale, reliability, validity, psychiatry

## Abstract

Background: Constipation is a functional gastrointestinal disorder that presents with signs and symptoms, which are typically assessed subjectively. Various measurement scales, such as the Constipation Assessment Scale (CAS), are commonly used to evaluate constipation among the general population. However, the instruments should be culturally and contextually relevant in adult psychiatric patients to generate valid and reliable evidence. Purpose: This study aimed to cross-culturally adapt and psychometrically validate the traditional Chinese version of the CAS among adult psychiatric patients in Hong Kong. Method: Using the Brislin protocol and Consensus-based Standards for the selection of health Measurement Instruments (COSMIN) guidelines, the CAS was translated into traditional Chinese and tested for internal consistency, test–retest reliability, content validity, and construct validity among psychiatric patients in Hong Kong. Results: The CAS was successfully translated into CAS-TC. The CAS-TC version demonstrated good content validity (scale level CVI = 97%), internal consistency (Cronbach’s alpha = 0.79), and test–retest reliability (ICC = 0.722 [95% CI, 0.587–0.812]). The CAS-TC showed a two-factor loading for the construct validity, which explained 54% of the total variance. Conclusions: The CAS-TC is valid and reliable and can be employed to assess constipation among adult psychiatric patients.

## 1. Introduction

The American College of Gastroenterology defines constipation as unsatisfactory defecation, characterized by infrequent stools, difficult stool passage, or both, for at least three months [1] (Ford et al., 2014). Constipation is a common problem that affects about 10% of the general population, 40% of individuals over 65 years of age, and more than 50% of individuals living with cancer [2]. Constipation is also prevalent among psychiatric patients; 20.3% of 4728 patients with schizophrenia had constipation [3], and 36.3% of patients with psychiatric diagnoses received at least one medication for constipation [4]. A meta-analysis of 32 studies showed that the prevalence of clozapine-related constipation was 31.2% (95% CI: 25.6–37.4) [5]. Moreover, psychiatric patients often have relatively more severe symptoms of constipation when they are found/reported. Antipsychotic drugs (such as clozapine) may cause anticholinergic side effects that cause constipation and are also accompanied by an increased threshold for discomfort or pain, which can be why these patients report somatic complaints in a delayed manner or less frequently [6]. Negative symptoms of schizophrenia are also associated with more indifference and problems with appropriate expressions of discomfort or pain sensations [6,7].

Constipation presents various signs and symptoms such as abdominal bloating, straining, difficulty passing stools, incomplete evacuation, hard lumpy stools, and prolonged time to pass stools [1]. Diagnosis of constipation is made in clinical settings using the Rome III criteria, which are commonly used in research and clinical practice [8]. The Rome III criteria hold that for a diagnosis of constipation, the patient should present with these conditions: (i) loose stools are rarely present without the use of laxatives, (ii) insufficient criteria for irritable bowel syndrome, and in addition, (iii) the patient should have two or more of the following symptoms: straining at defecation for at least a quarter of the time; lumpy and or hard stools for at least a quarter of the time; a sensation of incomplete evacuation for at least a quarter of the time; sensation of anorectal obstruction/blockage for at least a quarter of the time; manual maneuvers to facilitate defecation at least a quarter of the time; and three or fewer defecations per week [1]. All symptoms must have occurred for at least three months or more, with the onset of symptoms having occurred more than six months prior to diagnosis [1,8].

To evaluate the success of interventions aimed at alleviating and treating constipation, outcome measurement scales such as patient-reported instruments are commonly used. The subjective assessment of constipation usually involves using either generic or patient-specific self-reported outcome measures [9]. Various constipation evaluation questionnaires are reported in the literature, including the Bristol stool chart [10] the Constipation Assessment Scale [11], the Patients with Fecal Incontinence & Constipation Questionnaire [12], the Subjective Bowel Function Questionnaire [13], the Constipation Scoring System [14], and the Patient Assessment of Constipation Symptoms [15]. The Bristol stool chart is designed to classify the form of human feces into seven categories, which is more about the stool types, and is therefore not suitable for assessing constipation [11]. Among the other instruments, the Constipation Assessment Scale, in particular, includes the frequency of bowel movement, which is an important but non-universal indicator of constipation [16,17,18].

The CAS is based on the operationalization of constipation which is consistent with the definition given by the American College of Gastroenterology. In addition, the CAS is straightforward and easy to complete (around two minutes). The CAS is an eight-item patient self-report instrument, which asks questions about abdominal distention or bloating, change in the amount of gas passed rectally, less frequent bowel movements, oozing liquid stool, rectal fullness or pressure, rectal pain with bowel movements, the small volume of stool, and inability to pass stool [19]. Each item of the CAS is rated based on the patient’s experience with each characteristic in the previous week. The items are scored on a three-point summated rating scale (0 = ‘no problem’, 1 = ‘some problem’, 2 = ‘severe problem’). The final score of the CAS ranges from 0 (no constipation) to 16 (severe constipation) [19]. Moreover, CAS is used to evaluate constipation among various groups of people, including cancer survivors and pregnant women [20]. The Constipation Assessment Scale (CAS) was originally designed to evaluate the presence and severity of constipation in cancer patients [19]. The CAS was further modified and validated in a sample of pregnant women. The CAS demonstrated satisfactory internal consistency (Cronbach’s α = 0.70), good test–retest reliability (r = 0.98, *p* < 0.05), and significant contrasts between cancer patients with constipation and healthy controls (t = 6.32, *p* < 0.001) [11]. Despite having universal validity testing and applications in diverse patient groups, the CAS has not been studied in Chinese cultural settings and is limited when measuring constipation among psychiatric patients, which may present differently from cancer patients or pregnant women.

To conclude, a robust constipation evaluation scale for Chinese patients with psychiatric diagnoses is lacking. Hence, this study aimed to cross-culturally adapt and psychometrically validate the CAS among adult psychiatric patients of traditional Chinese (TC) language users in the Hong Kong Special Administrative Region of China. Specifically, the CAS was translated into TC and tested for content validity, internal consistency, test–retest reliability, and construct validity among adult psychiatric patients.

## 2. Methods

### 2.1. Design

This study employed cross-cultural adaptation and psychometric validation design. The cross-cultural adaptation and psychometric validation consisted of two interrelated phases: translation and validation, followed by psychometric evaluation [21,22]. The original CAS was translated into TC and assessed for content validity in the first phase. The following psychometric properties were assessed in the second phase: internal consistency, inter-rater reliability, and construct validity.

Phase 1: The Translation Process.

The essence of the translation is to produce an equivalent instrument in the target language, TC in this case. For this reason, the Brislin model of translation was employed to translate the CAS into TC, as follows [23]. Initially, two bilingual research nurses independently translated the original English CAS into the TC language. The translated TC version was then discussed among the two translators and an arbitrator to find and amend the differences or disagreements on items. The amended TC version of CAS was then backward translated into English by another bilingual translator. The back-translated CAS was compared with the original CAS by the researchers. Any significant differences in the meaning of the items were clarified, and their corresponding Chinese items were amended as needed. Subsequently, the initial TC version of CAS was developed, the CAS-TC. All measures for obtaining equivalence were observed to attain cross-cultural relevance [24,25,26].

Phase 2: Psychometric Validation Process.

### 2.2. Participants

A convenience sampling technique was employed by a research assistant to recruit the participants in a regional psychiatric hospital in Hong Kong. The sample inclusion criteria were (1) adult psychiatric in-patient (age 18–64), (2) ability to read and speak TC, and (3) ability to understand the questionnaires. Inpatients who presented with acute illness such as fever or active infectious disease and were mentally unstable, as suggested by their attending psychiatrist, were excluded. The sample size was determined based on the power analysis for the statistical test, which was the Exploratory Factor Analysis (EFA) involved in this study. As suggested by Nunnally (1978), the estimated sample size for the EFA is at least 10 participants per item [27]. For the eight items of the CAS and considering a possible 10% attrition rate, 90 subjects were recruited.

### 2.3. Study Setting

The study was carried out in an adult psychiatric team of a regional psychiatric hospital in Hong Kong, consisting of two admission wards, two rehabilitation wards, and one long-term care ward.

### 2.4. Ethical Considerations

The ethics application was approved by the Human Subjects Research Ethics Sub-Committee at The XX University and the Clinical Research Ethics Committees of the Hospital Authority, Hong Kong. Informed consent was obtained from each participant by an independent research assistant. The participants were voluntary and could withdraw without affecting their treatment or relationship with service providers. All data is anonymized and will be stored on a password-protected computer. All researchers signed nondisclosure agreements.

### 2.5. Outcomes

#### 2.5.1. Content Validity

To test the content validity of the CAS-TC, the appropriateness of all items in the CAS-TC was reviewed by a panel of ten experts for content validity, comprising two rehabilitation nurse consultants, one urological nurse specialist, three surgical nurse specialists, two advanced practice psychiatric nurses, and two nurse educators [28].

#### 2.5.2. Internal Consistency and Test–Retest Reliability

The internal consistency and test–retest reliability of the CAS-TC were assessed using the consensus-based standards to select health status measurement instruments (COSMIN) criteria [29]. In order to avoid recall bias and change in constipation condition, retest had been performed after five days of the first test.

#### 2.5.3. Construct Validity

The construct validity of the translated CAS-TC was examined using EFA analysis based on the COSMIN criteria [29].

### 2.6. Data Collection Procedure

A set of self-administered questionnaires, consisting of a demographic and clinical data sheet, Rome III criteria of constipation, and the CAS-TC were distributed to the consented participants individually. The patients filled these questionnaires in a quiet place with a significant distance apart from each other to minimize any influence from other participants. The participants were allowed to ask for help or explanation whenever necessary. The demographic and clinical data sheet was collected by checking the medical record system. Specifically, the following demographic information was included: patients’ gender, age, body mass index duration of constipation, self-medication for constipation, and predisposing factors for constipation such as physical exercise, fiber and fluid intake, and habit of defecation. In addition, clinical information about psychiatric diagnosis, duration of illness, type and dosage of medication, treatment for psychiatric illness, and treatment for constipation such as laxatives and its dosage were also collected. The participants were asked to complete the CAS-TC again at four days intervals. The time frame between the first and the second data collection was long enough to eliminate the memory effect; however, short enough to avoid any significant changes in symptoms of constipation that the subject experienced (the CAS only measures the symptoms of constipation in the past three days).

### 2.7. Data Analysis

Descriptive statistics of frequencies, means and standard deviations (SD) were used to summarize the participants’ demographic and clinical data.

The content validity of the CAS-TC was evaluated by a panel of ten experts on a 4-point Likert scale (4 = very relevant, 3 = quite relevant, 2 = somewhat relevant, and 1 = not relevant).The experts provided feedback on areas for improvement and suggestions for new items during individual interviews with the research team [30]. Item-level content validity indexes (CVI) were calculated for each item and the overall scale. An overall scale CVI score of 80% or higher was considered as good content validity [26].

The reliability of the CAS-TC was determined using internal consistency and test–retest reliability. The internal consistency was measured by calculating Cronbach’s alpha. A Cronbach’s alpha value of or above 0.70 indicates acceptable internal consistency. The test–retest reliability was assessed using intra-class correlation coefficient (ICC), with a value of or above 0.70 considered acceptable [28,29].

The construct validity of the CAS-TC was evaluated using EFA. The EFA explored the latent factors underlying the symptoms and gathered the information collected for assessing the inter-relationships among items and the overall scale of the CAS-TC. Items that had a corrected item-total correlation < 0.3 and whose deletion caused an increase of 0.1 or more in the alpha coefficient for the overall scale were considered as non-homogenous and were dropped [28,29].

To enhance the validity of data analysis, data were checked before performing the analyses to eliminate any discrepancies between raw data and the entered data. The data were analyzed using the IBM SPSS version 20.0 (IBM SPSS Statistics, Armonk, NY, USA: IBM Corporation).

## 3. Results

### 3.1. Phase 1: Translation

The original CAS was translated to TC successfully. However, some adjustments were made to clarify ambiguous meanings and correct the ambivalent wordings. For instance, bowel movement (i.e., 腸蠕動) was changed to 排便; 一直想要大便卻不乾淨 (Defecation; always wanting to have a bowel movement but not getting it totally out), urge/inability to pass stool was changed to 急大便卻無法排出 (feel urgent for a bowel movement but unable to pass). The amended items, together with the translators’ comments, were presented to the expert committee for content validity rating. Subsequently, the process was repeated until all items achieved more than 80% appropriateness rating by the panel of experts. Finally, the translation produced an equivalent instrument that attains conceptual, item, semantic, and operational equivalence.

### 3.2. Phase 2: Psychometric Validation

#### Demographic Characteristics

A total of 108 individuals met the inclusion criteria and responded to the study with valid written informed consent. The majority of the participants were males (N = 89), and nearly half of the participants (N = 53) were aged 35–52 years. Most of the participants were overweight (N = 64) and presented with schizophrenia (N = 95). In addition, more than two-thirds of the participants (N = 82) had a duration of illness over 24 months. Nearly all participants (N = 104) were taking psychotropic drugs (specifically antipsychotics and antidepressants), and a sizeable number of the participants (N = 40) did not experience constipation prior to taking psychotropic drugs. Detailed demographic and clinical characteristics of the participants are summarized in Table 1 below.

### 3.3. Content Validity

Through repeated adjustments, the item-level CVIs for the CAS-TC ranged from 87.5% to 100%, and the scale level CVI was 97%, which is greater than 80%, indicating good content validity. All eight items of the CAS-TC were rated as very relevant in terms of culture and content by all members of the panel, except item 4, ‘oozing liquid stool’ 滲便 (有大便從肛門滲出來) and item 5, ‘rectal fullness or pressure’ 便後有殘便感 (便後仍有大便的感覺) that received 87.5% and 90.0%, respectively. The calculated percentage of the appropriateness of the items is presented in Table 2 below.

### 3.4. Reliability

The internal consistency of the eight items of the CAS-TC had a Cronbach’s alpha of 0.791 for the total scale (See Table 3). The inter-item correlation of all paired-up items ranged from 0.07 to 0.55 (See Table 4). All the positive values showed that the items were measuring the same dimension, and it indicated that item two (change in the amount of gas passed rectally) and item three (less frequent bowel movements) had the highest correlation. In contrast, the lowest correlation was found between item four (oozing liquid stool) and item five (rectal fullness or pressure). Moreover, the mean of inter-item correlations was 0.39.

Among the 108 participants for test–retest reliability, 7 participants dropped out due to discharge or home leave and could not be reached during the retest period. The test–retest reliability of the CAS-TC was satisfactory, with a Cronbach’s alpha of 0.749 for the total scale and ICC of 0.722 (0.587–0.812) at four days intervals. The test–retest reliability shows a significant correlation between the two measurements.

### 3.5. Construct Validity

Bartlett’s test of Sphericity and the index of Kaiser-Meyer-Olkin of the CAS-TC were *p* < 0.05 and 0.774, respectively. This indicated that the data collected had high factor-ability and was applicable for factor analysis. Principal factor extraction with Varimax rotation was performed on all items of the CAS-TC for the total sample (n = 108). Two factors were extracted with Eigenvalues greater than one, explaining 54.51% of the total variance.

Based on the principal extraction result, less frequent bowel movements, oozing liquid stool, rectal fullness or pressure, rectal pain with bowel movement and urge but an inability to pass stool could be grouped into factor one. While abdominal distension or bloating, change in the amount of gas passed rectally, and small stool size could be categorized into factor two. The Eigenvalues for factor 1 and factor 2 were 3.35 and 1.01, respectively. However, the criterion for dividing the dimensions of the scale was that the loading factor should be greater than 0.7. Most (5/8) of the loading coefficients in this study are less than 0.7. The Cronbach’s alpha of factor 1 and factor 2 were less than 0.7. In addition, we added the EFA result in the Supplementary Material S1. Based on Factor one, the average variance extraction value is 0.295, which is less than 0.5, and the combined reliability CR value is 0.653, which is less than 0.7. Based on factor two, the average variance extraction value is 0.402, less than 0.5, and the combined reliability CR value is 0.668, less than 0.7, indicating that the extraction degree of measurement indicators in these two factors is poor.

Finally, this study complied with the original scale and kept one scale without dividing it into two dimensions. Please find more details in Table 5 and Supplementary Material S1.

## 4. Discussion

This study aimed to cross-culturally adapt the CAS into TC and assess the CAS-TC for internal consistency, test–retest reliability, content validity, and construct validity among patients with a psychiatric diagnosis. The findings of the study indicated that the CAS-TC was successfully translated and adapted to TC. Moreover, the CAS-TC demonstrated good psychometric properties. Hence, the instrument is valid and reliable among a group of TC speakers with a psychiatric diagnosis.

The overall objective of cross-cultural adaptation is to produce an equivalent instrument in the target culture [31]. Different conceptualizations and dimensions of equivalence were evident in the literature [32,33]. Based on the universalist approach, the concept of equivalence comprises five inter-related aspects: conceptual, item, semantic, operational, and functional equivalence [31]. Conceptual equivalence is concerned with the meaning and importance of the domain of the instrument in both the primary and target culture. The item equivalence shows that in both the primary and the target culture, the items of the instruments are acceptable and valid. On the other hand, the semantic equivalence denotes that the meaning of the scale items is the same in both cultures [31].

Similar to the original CAS, the CAS-TC demonstrated relevant and acceptable conceptual, item, and semantic equivalence in the TC culture, based on the robust translation employed, followed by the panel of expert evaluation of the CAS-TC. The Brislin method used in the translation of the CAS is a common technique for the translation and adaptation of patient-reported outcome measures, and the iterative steps ensure that a culturally equivalent instrument is produced [34]. The items of the CAS-TC were deemed meaningful, relevant, and culturally appropriate by the panel of experts, with all the items scoring 100%, except two, item four, ‘oozing liquid stool’ 滲便 (有大便從肛門滲出來) and item five, ‘rectal fullness or pressure’ 便後有殘便感 (便後仍有大便的感覺) that received 87.5% and 90.0%, respectively. This lower performance of the items could be due to cultural variations between people that speak English and TC. The items may be seen as culturally inappropriate by some individuals and lack similar expression in the TC culture. For example, Chinese culture emphasizes interpersonal relatedness (i.e., collectivism), and Western culture highlights the concern with one’s own needs, goals, and interests than with group-oriented problems (i.e., individualism) [35]. Meanwhile, bowel movements are viewed specifically by Chinese people as the depletion of Yin, which may result in insufficient intestinal fluids, and this is at odds with the oozing liquid stool item [36,37].

The operational equivalence denotes that the scale can be used the same way as in the primary culture [31]. Since constipation is a universal experience, the CAS-TC could be employed to evaluate constipation among TC speakers [1]. The measurement equivalence is achieved when the adapted instrument has the same psychometric performance as the primary instrument [31]. Previous studies have demonstrated that the original CAS is valid and reliable in the general population and pregnant mothers [11]. Similarly, this study has shown that the CAS-TC is valid and reliable in a sample of psychiatric patients. On the other hand, the functional equivalence is the summation of the proceeding equivalence, which indicates that both versions of the instruments do exactly what they were designed to achieve. In this case, both the CAS-TC and original CAS measure the same construct of constipation [31].

In comparison with extant literature, the findings of the present study are consistent with what was reported earlier in Italian [38] and Turkish [39] populations, where the CAS was cross-culturally adapted and psychometrically validated. Although we tried to explore whether different dimensions were available for this translated version of the scale, the EFA and CFA result suggested keeping it unidimensional, so we kept the original scale arrangement. However, there are some differences. For instance, in the Italian study [38], the construct validity was assessed using a known group validity method. Moreover, the population studied was a sample of apparently healthy individuals and palliative care patients. On the other hand, the Turkish study was conducted solely among apparently healthy students. The Turkish version was evaluated for construct validity using both exploratory and confirmatory factor analysis [39]. However, the current study population is people with severe mental illness. The cultural difference between the Italian and the current study may lie in that the Italian version focused more on privacy [38]. They thought the CAS could be self-completed by patients, which provides them with greater privacy. Consistent with this study, the Italian version also mentions that the tool’s strengths lie in its simplicity and speed of completion and suggests that physically weak patients with self-reported difficulties can complete CAS with the help of a nurse.

The findings of the present study should be interpreted with caution due to inherent study limitations. Specifically, the sample studied was recruited using a convenience sampling approach and in a group of psychiatric patients who were mostly middle-aged men, thus limiting the generalizability of the results. Secondly, the floor and ceiling effect of the CAS-TC was not evaluated. Hence, the attenuation effect of the CAS-TC could not be established. However, this study employed a robust translation protocol to attain cross-cultural equivalence, and the sample size used was large enough to assess the reliability and validity of the CAS-TC.

## 5. Conclusions

This study aimed to cross-culturally adapt and psychometrically validate the CAS among the TC-speaking population with a psychiatric diagnosis. The CAS was successfully translated and adapted into TC, thereby producing an equivalent instrument. The CAS-TC demonstrated acceptable internal consistency, test–retest reliability, content validity, and construct validity. Hence, the CAS-TC can be used in clinical practice among TC speakers. Future studies should employ a larger sample size with a clear gender balance to minimize bias and establish robust evidence. Moreover, further validation of the CAS-TC among the general population is warranted.

## Figures and Tables

**Table 1:** Demographic and clinical characteristics of the participants (N = 108).

Characteristics	N (%)
Gender	
Male	89 (82)
Female	19 (18)
Age	
18–34	27 (25)
35–52	53 (49)
53–65	28 (26)
BMI	
<18.5	3 (3)
18.5–23.9	41 (38)
>24	64 (59)
Diagnosis	
Schizophrenia	95 (88)
Depression	5 (5)
Bipolar disorder	4 (4)
Psychotic disorder	4 (4)
Duration of mental health condition	
<3 months	13 (12)
3–12 months	9 (8)
12–24 months	3 (3)
>24 months	82 (76)
Comorbidity	
Diabetes	9 (8)
Hypertension	4 (4)
Heart disease	1 (1)
None	94 (87)
Psychotropic drugs	
Yes	104 (96)
No	4 (4)
Constipation history	
Before taking psychotropic drug	40 (37)
After taking psychotropic drugs	83 (77)
Frequency of defecation	
<3 times a week	63 (58)
>3 times a week	45 (42)
Pattern of defecation	
Irregular	48 (44)
Delayed	32 (30)
Use of laxatives	
Yes	28 (26)
No	80 (74)
Adequate fiber intake	
Yes	38 (35)
No	70 (65)
Water intake (over 1500 mL per day)	
Yes	60 (56)
No	48 (44)
Exercise performance (at least 150 min of Moderate to Vigorous activity per week)	
Yes	55 (51)
No	53 (49)

**Table 2:** Appropriateness of the scale items.

		Items							
		1	2	3	4	5	6	7	8
Experts	1	4	4	4	4	4	4	4	4
	2	4	4	4	4	4	4	4	4
	3	4	4	4	3	3	4	4	4
	4	4	4	4	3	4	4	4	4
	5	4	4	4	4	4	4	4	4
	6	4	4	4	3	4	4	4	4
	7	4	4	4	4	3	4	4	4
	8	4	4	4	4	3	4	4	4
	9	4	4	4	3	3	4	4	4
	10	4	4	4	3	4	4	4	4
Percentage (%)		100	100	100	87.5	90	100	100	100

Footnote: Items: (1) Abdominal distension or bloating, (2) Change in the amount of gas passed rectally, (3) Less frequent bowel movements, (4) Oozing liquid stool, (5) Rectal fullness or pressure, (6) Rectal pain with bowel movements, (7) Small stool size, and (8) Urge but the inability to pass stool.

**Table 3:** The reliability and test-reliability of CAS-TC.

Items	Test Period (N= 101)		Test–ReTest Period (N = 101)		r	*p*
	Mean (SD)	Cronbach’s Alpha	Mean (SD)	Cronbach’s Alpha		
1. Abdominal distension or bloating	0.32 (0.58)		0.42 (0.59)		0.415	0.001 ***
2. Change in amount of gas passed rectally	0.44 (0.58)		0.47 (0.61)		0.279	0.005 **
3. Less frequent bowel movements	0.37 (0.57)		0.32 (0.51)		0.270	0.006 **
4. Oozing liquid stool	0.21 (0.49)		0.25 (0.48)		0.343	0.001 ***
5. Rectal fullness or pressure	0.52 (0.56)		0.49 (0.58)		0.349	0.001 ***
6. Rectal pain with bowel movement	0.29 (0.53)		0.28 (0.53)		0.523	0.001 ***
7. Small stool size	0.31 (0.51)		0.40 (0.57)		0.342	0.001 ***
8. Urge but inability to pass stool	0.37 (0.59)		0.45 (0.61)		0.427	0.001 ***
Total score	2.83 (2.82)	0.791	3.05 (2.70)	0.749	0.565	0.001 ***

Footnote: *** *p* < 0.001; ** *p* < 0.01.

**Table 4:** The inter-item relevance matrix of CAS-TC.

	Item 1	Item 2	Item 3	Item 4	Item 5	Item 6	Item 7	Item 8	Total Score
Item 1		0.354	0.509	0.280	0.229	0.182	0.448	0.248	0.643
Item 2			0.546	0.097	0.335	0.407	0.418	0.369	0.703
Item 3				0.181	0.154	0.354	0.433	0.420	0.716
Item 4					0.071	0.335	0.291	0.112	0.442
Item 5						0.283	0.346	0.122	0.498
Item 6							0.497	0.464	0.683
Item 7								0.515	0.766
Item 8									0.645

Footnote: Items: (1) Abdominal distension or bloating, (2) Change in the amount of gas passed rectally, (3) Less frequent bowel movements, (4) Oozing liquid stool, (5) Rectal fullness or pressure, (6) Rectal pain with bowel movements, (7) Small stool size, and (8) Urge but the inability to pass stool.

**Table 5:** Principal factor extraction and factor loadings of each item of CAS-TC.

	Factor 1	Factor 2	Cronbach’s Alpha
Rectal pain with bowel movement	0.763	0.122	
Oozing liquid stool	0.708	−0.099	
Urge but inability to pass stool	0.674	0.392	
Less frequent bowel movements	0.531	0.489	
Rectal fullness or pressure	0.507	0.262	0.626
Change in amount of gas passed rectally	−0.169	0.854	
Small stool size	0.451	0.532	
Abdominal distension or bloating	0.279	0.478	0.668
Eigenvalue	3.35	1.01	
Cumulative variance contribution rate	35.58%	54.51%	

## Data Availability

The data presented in this study are available on request from the corresponding author.

## Supplementary Materials

The following supporting information can be downloaded at: https://www.mdpi.com/article/10.3390/ijerph20032703/s1. Supplementary Material S1 Confirmatory Factor Analysis Result.
