# Supplementary material for: Cross-Cultural Adaptation and Psychometric Validation of the Constipation Assessment Scale among Chinese Adult Psychiatric Patients
Source: Int J Environ Res Public Health. 2023 Feb 2;20(3):2703. doi: 10.3390/ijerph20032703 (PMC9915141; doi:10.3390/ijerph20032703)
Supplement: Supplementary file 1 [file ijerph-20-02703-s001.zip › ijerph-2124485-supplementary.pdf]

Supplementary Material S1 Confirmatory Factor Analysis Result

Table S1 Factor loading of each item of CAS-TC.

Factor	Items	Non-standard load factor	Standard load factor	z	S.E.	P
Factor 1	Rectal pain with bowel movement	1	0.772	-	-	-
	Oozing liquid stool	0.429	0.355	2.943	0.146	0.003***
	Urge but inability to pass stool	0.875	0.607	4.235	0.207	0.000***
	Less frequent bowel movements	0.733	0.523	3.957	0.185	0.000***
	Rectal fullness or pressure	0.42	0.309	2.608	0.161	0.009***
Factor 2	Abdominal distension or bloating	1	0.616	-	-	-
	Small stool size	1.033	0.728	3.754	0.275	0.000***
	Change in amount of gas passed rectally	0.943	0.574	3.946	0.239	0.000***

Table S2 The AVE and CR of proposed two factors of CAS-TC

Factor	AVE	CR
Factor 1	0.295	0.653
Factor 2	0.402	0.668

Notes: AVE, Average Variance Extraction CR, Construct Reliability
